# Enhancement Studies on Manufacturing and Properties of Novel Silica Aerogel Composites

**DOI:** 10.3390/gels4010005

**Published:** 2018-01-07

**Authors:** Sunil Chandrakant Joshi, Periyasamy Manikandan, Yogeswari Jothi

**Affiliations:** School of Mechanical and Aerospace Engineering, Aerospace Engineering cluster, Nanyang Technological University, 50 Nanyang Avenue, Singapore 639798, Singapore; manikandanp@ntu.edu.sg (P.M.); yogeswarijothi3007@gmail.com (Y.J.)

**Keywords:** aerogel composites, mechanical characterization, sandwich design, cyclic loading

## Abstract

Silica Aerogel composites are ultra-low density, highly porous foam-like materials that exhibit excellent thermal insulation and high strain recovery characteristics. In the present work, environment-friendly silica aerogel composites are fabricated using silica aerogel granules with bio based porcine-gelatin as the binding agent dissolved in water and by further drying the mix at sub-zero condition. This article focuses on improvement studies carried on the mold design and the manufacturing process to achieve better geometric compliance for the silica aerogel composites. It also presents contact angle measurements, compressive behavior under different cycles of loading, time dependent behavior and flexural response of the composites. The influence of additives, such as fumed silica and carbon nanotubes on mechanical properties of the composites is also deliberated. Water droplet contact angle experiments confirmed the ultra-hydrophobic nature of the composites. The mechanical properties were characterized under cyclic loading-unloading compression and three-point flexure tests. On successive compression in three consecutive load cycles, the strain and thickness recovery were found to decrease by around 30%. The flexural properties of the aerogel composites were investigated using it as the core covered by thin carbon composite face sheets. It was found that the flexural strength and the failure strain of this aerogel sandwich composites is approximately half of the conventional nomex honeycomb sandwich equivalent.

## 1. Introduction

Aerogels are a unique class of material that can be synthesized from either organic or inorganic precursors. Aerogels are renowned as the lightest solids, often called “solid smoke” because of their ultralow density. Among the available aerogel varieties, silica (SiO_2_) aerogels are more popular because, besides being highly porous offering large surface area, they exhibit low thermal conductivity, high optical transmission, low dielectric constant, low refractive index and low sound transmission speed. This unique combination of amazing properties makes silica aerogels useful in a wide range of sectors. One of the highly publicized applications was its usage in space experiments, where silica aerogel blocks are used to insulate electronics on MARS Rover and to collect space dust. They have also been used or considered for thermal insulation, pollution filters, dangerous liquid storage vessels, acoustic transducers, sound insulation, impact protection and many more [1].

Despite these peculiar features, the inherent fragility, poor mechanical properties and expensive production in usable sizes have restricted its application envelope. To alleviate these restrictions, innovative methodologies to develop mechanically robust and flexible silica aerogel solids or composites had been the focus of research. A comprehensive survey on recent developments of silica aerogel composites may be found in literature [2].

Noticeably, most of the explored proof-of-concepts are based on small-size samples, nothing bigger than a cubic centimeter cuboid and a small test tube size cylinder except the availability of some fiber reinforced thermal wrap aerogel blankets [3]. General physical and chemical issues entailed during the material synthesis were elaborately explored. However, manufacturing feasibility and difficulties associated when synthesizing large-size samples of regular geometry pattern are rarely documented. Indeed, this is very important for a new material while transforming it from conceptualization stage to realistic application stage. The present article addresses the difficulties associated during the manufacturing of large samples and possible measures taken to achieve an acceptably accurate final geometry within the current fabrication process described in [4].

Studies have shown that mechanical properties of silica aerogels are sensitive to multiple interdependent factors; such as synthesis chemistry, environment, storage, network connectivity and gel density [5]. The fragility of unreinforced native silica aerogels is primarily imparted by the presence of small neck connections present between the secondary silica particles and the existence of large stiff siloxane (Si–O–Si) bonds [2]. Different structural reinforcement approaches have been explored to dilute these stiff siloxane bonds and make aerogels not only flexible but also mechanically strong. It includes, (i) Synthesizing silica backbone through various flexible silica precursors [6,7] (ii) cross linking of different organic polymeric systems on the surface of silica back bone [8,9,10,11] and (iii) compounding of micro/nano fibers with silica back bone [12,13,14,15].

Mono-, di- or tri-functional organosilane compounds of the type R’_X_Si(OR)_4−X_ (where 1 ≤ X ≤ 3, R = alkyl, aryl or vinyl groups) are some of the appropriate sol-gel precursors vastly used for fabricating mechanically robust and flexible silica aerogel composites. Flexibility using these precursors is attributed to reducing the number of stiff Si–O–Si bonds with more flexible Si-R organic bonds [1]. Cross linking of several polymeric systems, such as polyuria, polyurethane, poly (methyl meta acrylate), polystyrene and polyacrylonitrile with native silica precursors have been found effective not only to increase flexibility, but also its mechanical strength increases by about three orders of magnitude [16]. The improvement in mechanical strength of silica aerogel is attributed to the strong interfacial covalent bond between the organic polymer phase and inorganic silica phase. Taking advantage of different reinforcement strategies, it is, in principle, possible to develop a flexible, mechanically strong and more versatile silica aerogel solid or composite. It is anticipated that the rise in aerogel composites applications is determined by two main factors; one, the level of ease to fabricate geometrically-accurate and compliant samples of large size and two, their structural integrity under complex mechanical and thermal loads.

With this background knowledge, the present research embarked on addressing some of the shortcomings in relatively new silica aerogel composites [4]. This article is structured into two different sections: first, the manufacturing aspects associated with flat aerogel composite samples, and second, a brief investigation on structural integrity and mechanical properties of the manufactured samples under cyclic compression and flexural loading conditions.

## 2. Results and Discussion

### 2.1. Influence of Mold Design

**Figure 1:**
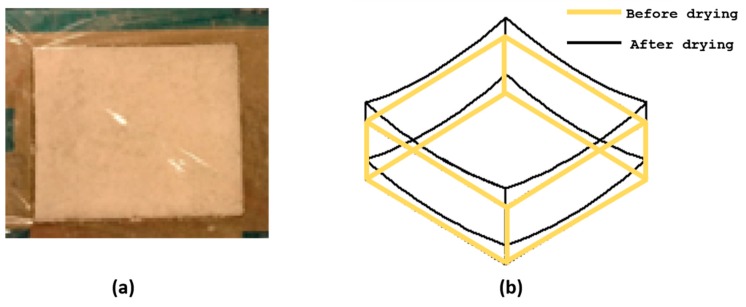
Original mold design, MDP00, and the resultant product (**a**) Top and bottom surface covered by porous transparent film (**b**) Observed synclastic form of curved aerogel sheet.

As shown in Figure 1, casting aerogel mixture on to the open square mold and covering its top surface only with a porous transparent ply (Figure 1a) resulted in a thin aerogel sheet, which was curved having a *synclastic shape* geometry instead of a perfect *flat shape* (Figure 1b) geometry upon vacuum freeze drying. This might be because of the absence of firm support on the top and bottom planar surfaces of the mold as well as non-uniform vacuum application on to the in-the-mold specimen. Several ideas have been put forth and a few were tried to modify the mold design in order to produce a perfectly flat aerogel composite sheets. This design and the process together are designated as MDP00.

In the first attempt, instead thin flexible ply, a thin polycarbonate sheet was placed on the top surface and firmly secured with the outer edge of the square mold using a tape. It was expected that this would provide a support against the vacuum pressure. In order to facilitate sublimation of water during the drying process under vacuum, tiny holes of 3 mm diameter were drilled at equal pitch over the entire surface of the polycarbonate sheet. Despite the top support given, the obtained aerogel samples still exhibited a curvature though its radius was smaller than the previous one. This design and the process together are designated as MDP01.

**Figure 2:**
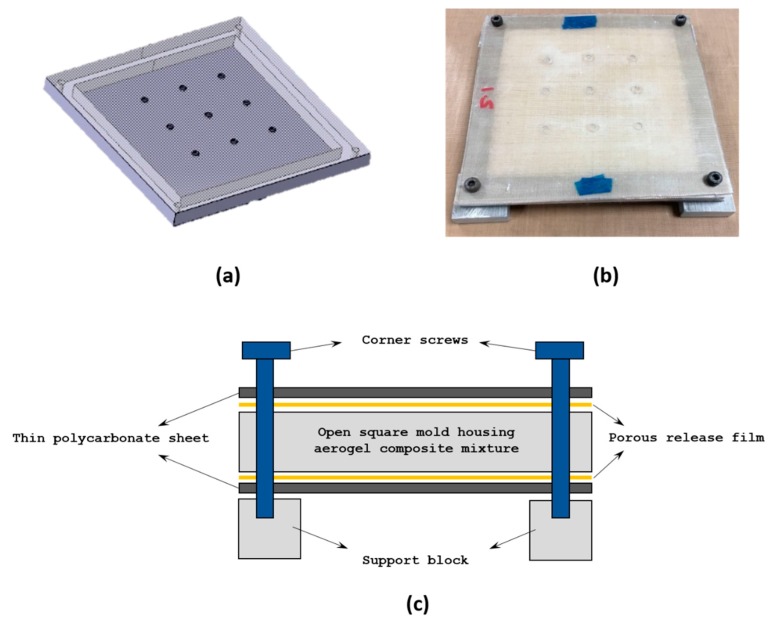
Proposed compact mold design MDP02 (**a**) Thin polycarbonate sheet having uniform holes over the surface (**b**) Assembled mold (**c**) Pictorial representation of assembled mold design.

In the second attempt, complete design of the square mold was altered. The polycarbonate sheets with uniformly drilled holes were placed on both top and bottom sides of the mold with the casting inside. The plastic sheets were fastened to the square mold frames on its four corners using screws as shown in Figure 2a,b. This arrangement also made it feasible to fabricate multiple samples in a single freeze-dry cycle through stacking a few molds one above the other inside the freeze dryer. To easily remove the dried aerogel samples without damaging its outer surface, a porous release film was placed between the aerogel casting and the plastic cover sheet as illustrated in Figure 2c. The tiny holes on the release film allowed the solvent within the composites to sublimate effectively under vacuum. Samples obtained using this mold design and the molding process resulted in perfectly flat specimen with no curvature or warpage on its top and bottom surfaces.

**Figure 3:**
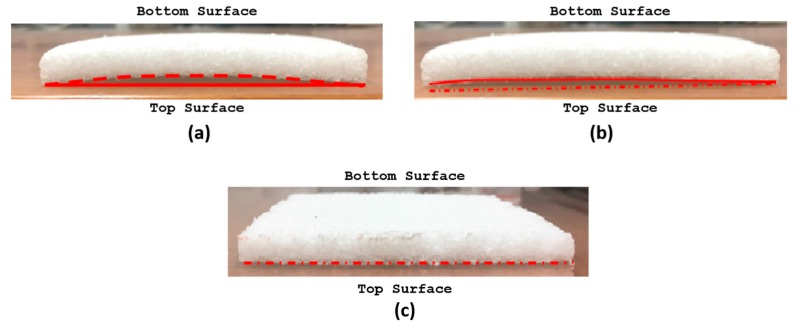
Side view of thin aerogel composite sheet obtained using different mold designs and process; (**a**) MDP00 (**b**) MDP01 (**c**) MDP02.

Figure 3 compares the resultant aerogel composite samples obtained from three designs, MDP00 to MDP02 experimented in this work. It is evident that placing a firm support sheet on both sides with proper channels for sublimation (i.e., MDP02) allows successful molding of thin aerogel composite flat sheets without any geometry problems or curvature issues.

This also highlights the need for extracting the solvent uniformly from all sides of the sample during the freeze-drying process. This evens out the local pressure distribution experienced by the material within the sample and maintains it that way throughout the process. As a result, the sample remains flat. This mold design and processing philosophy can be adopted and extended to fabrication of aerogel composites in any other non-flat or complex shapes. For thicker samples, it may also be required to allow sublimation through the thickness direction as well.

### 2.2. Hydrophobicity

**Figure 4:**
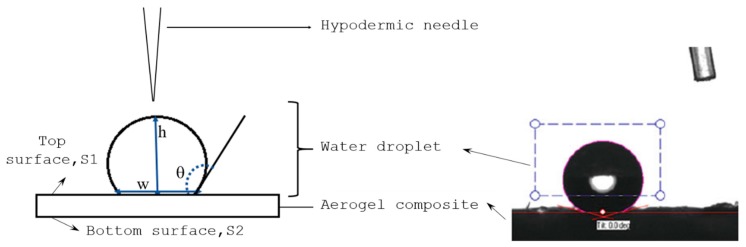
Contact angle measurements using water drop test.

Hydrophobicity of aerogel composites depends on the corresponding silica precursor, solvent and the drying method. It can be quantitatively measured using the contact angle (θ) of a water droplet on the aerogel composites surface, given by Equation (1) as;

(1) $$\theta =2\tan^{-1}\left(\frac{2h}{w}\right)$$

where, *h* and *w* are the height and width of the water droplet respectively as shown in Figure 4.

The contact angle of the sample Standard sample (SAC), sample compounded with fumed silica powder (FSC) and carbon nanotubes (CAC) were measured using ATOT automated instrument. The volume of droplet used to measure contact angle was auto-set to 5 μL. In order to have the representative surface roughness, the contact angle measurements are taken at 6 random points on the top, S1, and the bottom, S2, and their average value is considered in the further investigation. According to the contact angle results tabulated in Table 1, all the fabricated samples are hydrophobic in nature as their contact angles are greater than 90° even though there exists a slight variation between the top and bottom surface measurements. Such variation was probably induced by the difference in the surface condition that prevailed during freeze drying, where the bottom surface of sample was slightly smoother than the top surface. The reason could be the minor gravity effect by virtue of which the solvent droplets tend to accumulate near the bottom. As noticed, compounding with fumed silica powder (FSC) does not have significant influence on hydrophobicity compared to SAC. At the same time, addition of a tiny (0.08%) weight % of –OH functionalized CNT found to increase the contact angle by 10° on average. The reaction of –OH functional group present in CNT with the oxy-TMS (Tri Methyl Silicate) group on the aerogel surface makes the CAC samples more hydrophobic compared to the other counterparts [4].

### 2.3. Mechanical Properties of Silica Aerogel Composites

#### 2.3.1. Cyclic Compression Loading Behaviour

**Figure 5:**
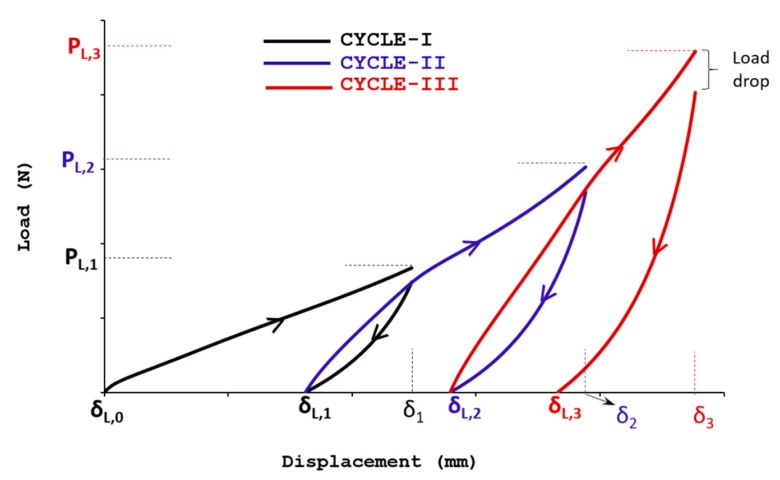
Schematic representation of cyclic compression loading/unloading test.

In order to study the sample response to cyclic compression, compressive load was applied by moving down the crosshead to compress the sample up to 25% of its thickness and then unloading to zero load by moving the crosshead up. The speed adopted was 1 mm/min. The permanent reduction in the thickness (%) was measured before commencing the next cycle. This process was repeated for three load-unload cycles for the same specified conditions. Figure 5 depicts the obtained typical uniaxial compressive response of the aerogel composite samples.

Where *δ* and *P* in Figure 5 denote the cross head displacement and force respectively. Suffix *i* to load *L* represents the corresponding loading parameters of *i*th cycle. From these tests, percentage change of peak load (ΔP) and recovered compressive deformation (Δδ) in the samples for each consecutive cycles are extracted using the relations (2) and (3) as,

(2) $$\Delta P_{i+1}=\left(\frac{P_{L,i+1}-P_{L,i}}{P_{L,i}}\right)\;x\;100$$

(3) $$\Delta \delta_{i}=\left(\frac{\delta_{L,i}-\delta_{L,i-1}}{\delta_{i}-\delta_{L,i-1}}\right)\;x\;100$$

The observed peak load and the respective percentage of peak load change for the successive compression load cycles are summarized in Table 2. As seen, the samples with greater amount of fumed silica content (FSC-80MG) and CNT (CAC) exhibit lesser compression resistance and a maximum of 10% reduction in the peak load was observed compared to SAC samples irrespective of the cycle number. On successive cyclic loading under same load condition, the peak load increment in all the sample found decreases, between cycle I and II the increment is around 75% but only around 45% recorded between cycle II and III for all tested aerogel variants.

Such behaviour implies that the compression resistance of aerogel composite samples reaches a asymptotic value on successive loading. Eventually the sample becomes a tightly packed thin sheet with negligible intrinsic air pockets compared to its original thick and foamy architecture. This may be further confirmed by the decreasing trend seen of the recoverable displacement of the samples on successive compression loading cycles, as summarized in Table 3. With increase in the number of cycles, the samples steadily lose their recoverability and the percentage of compressive displacement ($\Delta \delta_{i}$) retained during the unloading cycle was found to decrease from ~65% to ~40% for three number of loading/unloading cycles.

#### 2.3.2. Stress Relaxation Behaviour

Relaxation behaviour can be characterized by monitoring the stress as a function of time after the considered sample has been subjected to the specified compression loading. As seen in the cyclic compression loading/unloading response in Figure 5, there exists a notable load drop at the end of each loading cycle before the successive unloading. Similar response was noticed in other samples as well. Such behaviour justifies the relaxation tendency of foamy aerogel composite samples under axial compression loading.

To understand this behaviour in detail, the aerogel composite samples were compressively loaded up to 25% of their final thickness at the strain rate of 1 mm/min and the loaded sample was left undisturbed for 30 min without any further crosshead displacement. The load drop was recorded over the stated period of time and the associated compressive stress was used to estimate the relaxation nature of the aerogel composites.

**Figure 6:**
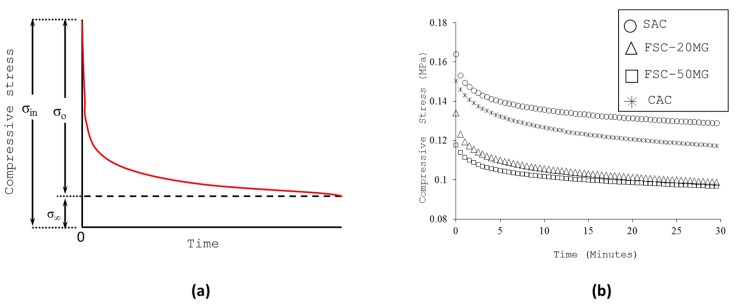
(**a**) Typical stress relaxation curve (**b**) Stress relaxation curve of aerogel composites.

The nature of the stress variation follows Kohlrausch function [17] of form described by Equation (4) given as,

(4) $$\sigma \left(t\right)=\sigma_{o}e^{-t^{b}}+\sigma_{\infty }$$

where σ(t) is the compressive stress at time t; $\sigma_{o}$ is the relaxing part of stress; $\sigma_{\infty }$ is the equilibrium part of stress; and b provide fractional exponent of time as typically illustrated in Figure 6a.

On dividing Equation (4) by the initial stress $\sigma_{in}$, it is feasible to relate the stress parameters in relative form as follows,

(5) $$K_{r}=K_{1}e^{-t^{b}}+K_{a}$$

where $K_{r}=\frac{\sigma \left(t\right)}{\sigma_{in}}$ is the relaxation coefficient; $K_{1}=\frac{\sigma_{o}}{\sigma_{in}}$ is the reduction factor, and $K_{1}=\frac{\sigma_{\infty }}{\sigma_{in}}$ is the attenuation factor. According to the experimental data shown in Figure 6b, the dependency of $K_{r}$ for the family of aerogel samples considered in the present study was found approximated by Equation (6),

(6) $$K_{r}\approx 0.23e^{-t^{b}}+0.75$$

The above number implies that the samples were relaxed by reducing their compression stress by 23% and conserve 75% of the remaining during the first 30 min. It is also noticed that the inclusion of other compounds didn’t influence the relaxation behaviour much for the FSC and CAC samples.

#### 2.3.3. Flexural Behaviour

Three point bending studies on aerogel composites provided the ultimate flexural strength of the composites. In order to avoid local indentations and crushing, one pre-cured carbon epoxy composite ply was used as facesheet with the aerogel composites as the core. The facesheets were only placed at the top and bottom of the core without any bonding. 

Assuming thickness of the face sheets as negligibly small, the flexural strength, $\sigma_{f}$, and strain, $\varepsilon_{f}$, can be calculated based on Euler Bernoulli Equations (7) and (8) [18],

(7) $$\sigma_{f}=\frac{3PL}{2bh^{2}}$$

(8) $$\varepsilon_{f}=\frac{6P\delta }{L^{2}}$$

where *P* is the maximum flexural load; *δ* is the displacement at the centre of beam, *L* is the span length, *b* and *h* are the width and thickness of flexural sample respectively. 

**Figure 7:**
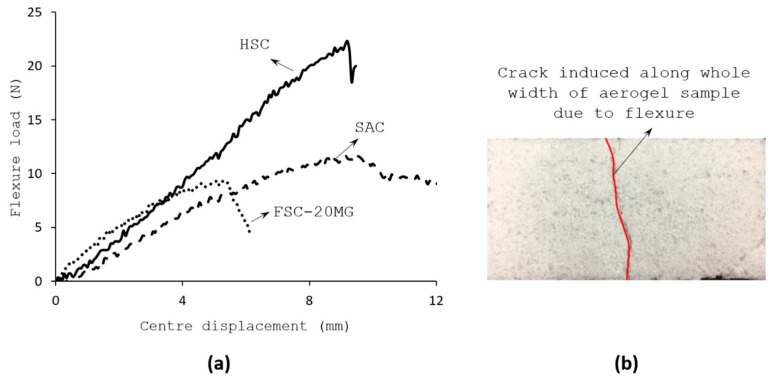
Results of three-point flexure test (**a**) Load-displacement curve (**b**) Tensile crack visible on bottom surface of aerogel composite.

Figure 7a shows the load- displacement curves obtained through three-point flexure test of SAC and FSC aerogel samples. In all cases, the abrupt drop in load was caused by the fracture of sandwiched core whilst the outer stiff CFRP face sheet layers remained undamaged. Regardless of the type of core reinforcement, all the tested sandwich samples exhibited a rather brittle behaviour in flexure, which fractured at the smaller displacement range. As noted, the thickness to displacement ratio is merely 1. As benchmark, the obtained results were compared with Nomex honeycomb core sandwich samples, HSC. Nomex honeycomb cores are made of aramid fiber paper impregnated with a phenolic polymer resin and it is one of the common core materials used to build load carrying sandwich structures [19]. FSC samples exhibited significant deterioration in the flexural ability of the aerogel composites. The displacement at failure for FSC sample was around 5.25 mm, which is 50% lower than the observed for the SAC sample. The failure appeared exactly under the loading pin where the crack is nucleated all along the width of the core in its bottom side and propagated towards the top side as the flexure progressed, as shown in Figure 7b.

The characteristic parameters obtained from the three-point flexural tests are summarized in Table 4. As noticed, for the same geometry and loading conditions, the SAC sample was the second best after the HSC samples in terms of the flexural response. The flexural strength and the failure strain of the SAC samples were 0.5 times of the HSC results.

## 3. Conclusions

Mold design iterations were envisaged and implemented so as to achieve a perfectly uniform, thin, and geometrically flat aerogel composites. The achieved samples using the altered mold design MDP002 resulted in a sample with no curvature or warpage. Having oxy-TMS (Tri Methyl Silicate) coating on the surface of silica granule precursors, all fabricated samples exhibited hydrophobic nature irrespective of the type of additive compounds used in hybrid variants. Mechanical behaviour of aerogel composite samples was characterized using uniaxial cyclic compression and three-point bending tests. On successive loading, the displacement recoverability was found to be decreased by 30% under the same load conditions for all tested composite variants. The flexural tests performed on the sandwich configurations where the aerogel composite was used as the core material sandwiched between thin carbon fiber polymer facesheets proved useful. Compared with conventional nomex honeycomb core sandwich samples, the aerogel based sandwich samples exhibited 50% flexure strength and failure strain. Despite these mechanical characterization tests, the fabricated aerogel composite samples are still under-qualified for load bearing applications. Further studies are required to add in flexibility and reduce brittleness of the aerogel composites. Notwithstanding, they are a good candidate for light weight hydrophobic applications less critical on load carrying functions. The influence of fumed silica and CNT additives to SAC samples was found to deteriorate mechanical response of the hybrid variants FSC and CAC and require further optimization studies.

## 4. Materials and Methods

### 4.1. Raw Materials

**Figure 8:**
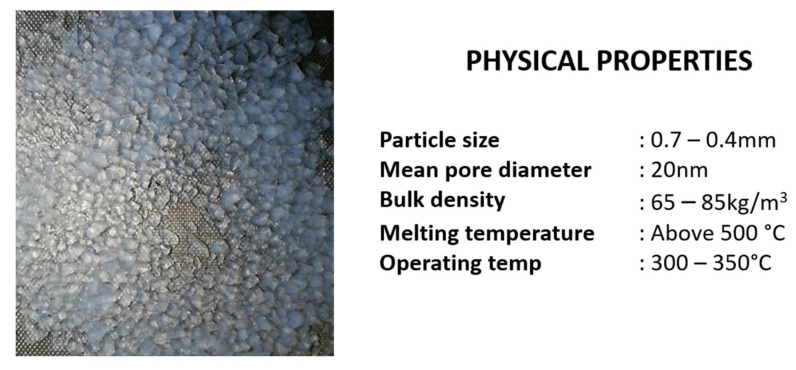
Silica aerogel granules used for composites fabrication and its physical properties.

Silica aerogel granules of size ranging between 0.1–4.0 mm, procured from Cabot Corp. (Boston, Massachusetts, USA) [20] were used as a precursor to fabricate the composites. The surface of these granules has oxy-TMS (Tri Methyl Silicate) groups attached making it hydrophobic. Physical appearance of aerogel granules and its basic physical properties are shown in Figure 8. Porcine-gelatin, a water soluble biocompatible polymer, acquired from Sigma Aldrich [21] was used as the binder with water as the solvent. Sodium Dodecyl sulphate (SDS), also from Sigma Aldrich, [22] was used as the foaming agent to facilitate mixing of the binder, solvent and the precursor granules. Additives, such as, fumed silica powder (SiO_2_) of 0.007 μm from Sigma Aldrich [23] and –OH group functionalized multi-walled carbon nanotubes (MWCNT) purchased from Nanostructured and Amorphous, Inc. (Houston, TX, USA) were added to the list of ingredients to check the influence they have on the resultant mechanical properties. Detailed properties of above stated materials were found in Reference [4].

### 4.2. Manufacturing Process

**Figure 9:**
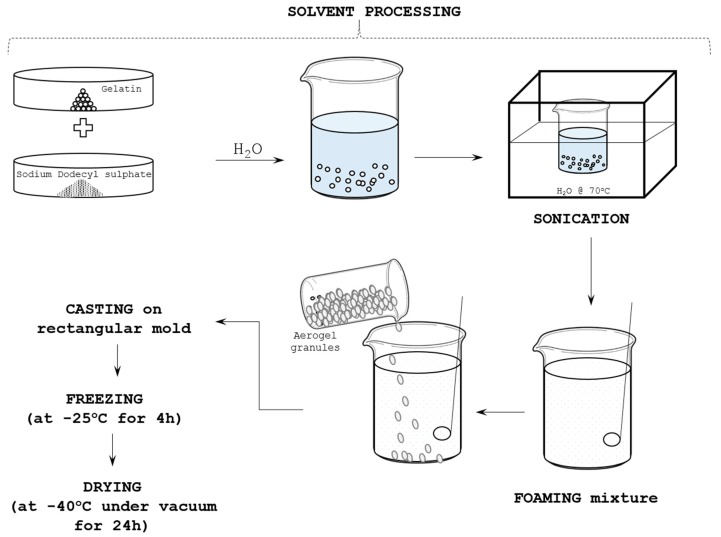
Schematic representation of silica aerogel composites synthesis procedure.

Figure 9 illustrates the basic aerogel composites manufacturing process [4] adopted in the current study. Overall it constituted of two major steps; (i) Preparation of colloidal aerogel mixture, and, (ii) freeze drying (FD) process. As stated in [4], first a pre-weighed quantity of 2.4 g of Gelatin and 0.05 g of SDS were mixed with 24 mL of H_2_O. The mixture was sonicated using Fisher Scientific FB15051 sonicator for 30 min to remove the dissolved gases in the mixture, followed by 45 min sonication at 70 °C to achieve complete dissolution of gelatin and SDS in H_2_O. The mixture is then stirred gently using electric stirrer for about 10–15 min until it turned thick and frothed. A pre-weighted quantity of about 9.5 g aerogel granules was then added into the frothed mix and stirred gently for 10–15 min until a paste-like mixture coagulated.

The obtained mixture was spread into a rectangular mold of 10 cm × 10 cm × 10 cm dimensions. It was covered using porous transparent release sheets, which allowed the H_2_O to sublimate under vacuum. Then, the mold with the casting inside is kept at −25 °C in a refrigerator for 3–4 h for the gelatin solution to freeze and stabilize. Similar technique of bio-based porcine-gelatin incorporation as a binder in Poly (Vinyl Alcohol) (PVA) solvent has been succeeded to develop excellent flame retardant PVA/Clay aerogels using freeze drying technique [24]. The frozen sample and the mold were then transferred to a freeze dryer to dry under vacuum for 18–24 h. This process dries almost the entire moisture from the sample. The temperature in the dryer starts to drop gradually until it reaches −45 to −48 °C which facilitates sublimation of the H_2_O. The obtained samples through this method are designated hereafter as standard aerogel composites samples (SAC).

To investigate improvements in the physical and mechanical properties of aerogel composites, additives compatible with the silica aerogel particles were tried out with the SAC samples. The inclusion of the fumed silica (denoted as FSC) made the SAC samples more compact with negligible increase in their weight. In the present study, samples were fabricated using two weight proportions of FSC; 20 mg and 80 mg. The other hybrid variant was fabricated by adding 0.08% weight of –OH functionalized multi-walled carbon nanotubes (designated as CAC). The in-situ reinforcement of CNT in to the SAC samples was believed to improve not only the mechanical performance but also the overall structural Integrity of the composites. These additives were added in to the solvent mixture during the mixing stage after the sonication. The details of the samples fabricated and tested in the present work are summarized in Table 5.

### 4.3. Characterization Methods

The hydrophobic nature of the aerogel composites was investigated by water droplet experiment using Attension Theta Optical Tensiometer (ATOT). ATOT is automated equipment which provides precise measurements of the static contact angle with a sessile drop to dispense water droplet, considering the 3D surface roughness, surface free energy, surface and interfacial tension and interfacial rheology of the material tested. The complete arrangement was maintained at a temperature of 23 ± 2 °C and relative humidity kept greater than 50%.

**Figure 10:**
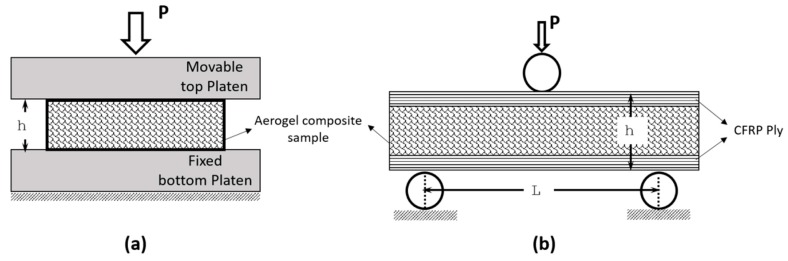
Mechanical characterization test (**a**) Compression test (**b**) Three-point flexure test.

The INSTRON 5569 Universal Testing Machine was used for mechanical property test at room temperature including uniaxial compression and three-point flexural test as shown in Figure 10. In the uniaxial compression test, the as-prepared 10 cm side length and 1 cm thick square samples were consecutively loaded and unloaded by maximum of three times. Such test allows measuring the volume reduction level pronounced in the aerogel samples due to repeated compressive loading. Furthermore, the stress relaxation behaviour of foamy aerogel composites was measured in the samples that were compressively loaded in the first cycle. Load decrement history was recorder for the period of 30 min which provide rate of relaxation exhibited by the aerogel composites.

The flexural responses of aerogel were measured by conducting three-point bending test on aerogel samples of size 10 cm × 5 cm × 1 cm sandwiched between carbon fiber reinforced polymer (CFRP) ply of 0.125 mm thickness. The reinforcement of thin CFRP layer on the outer surface persuaded the aerogel composites to freely flex and eventually fail by flexure. The strength obtained from three-point test provides an estimate of maximum flexural (also tensile) ability of aerogel composite material when it has been used as insulating layer between thin outer covering layers.

## Figures and Tables

**Table 1:** Measured contact angle.

Sample	θ (Degrees)		Remarks
	S1	S2	
SAC	110.1 ± 4	98.2 ± 6	All variants are hydrophobic.
FSC-20MG	108.0 ± 8	98.0 ± 5	
FSC-80MG	104.2 ± 3	103.2 ± 2	
CAC	117.5 ± 3	114.2 ± 1	

**Table 2:** Load magnitude from cyclic compression test.

Sample	Peak Load in Each Cycle (N)			% Peak Load Change		Young’s Modulus (kPa)
	*P_L_*_,1_	*P_L_*_,2_	*P_L_*_,3_	Δ*P*_2_	Δ*P*_3_	
SAC	1820 ± 280	3155 ± 510	4495 ± 600	73.3	42.5	750
FSC-20MG	1820 ± 230	3267 ± 405	4796 ± 620	79.5	46.8	774
FSC-80MG	1677 ± 160	3034 ± 325	4587 ± 95	80.9	51.2	691
CAC	1692 ± 80	2979 ± 450	4336 ± 235	76.1	45.6	690

**Table 3:** Displacement recovery from cyclic compression test.

Sample	Recovered Compressive Displacement, mm		
	Δ*δ*_1_	Δ*δ*_2_	Δ*δ*_3_
SAC	64 ± 4	49 ± 4	42 ± 4
FSC-20MG	66 ± 10	50 ± 11	42 ± 10
FSC-80MG	63 ± 7	52 ± 7	44 ± 1
CAC	64 + 14	49 ± 12	41 ± 14

**Table 4:** Characteristic parameters from three-point flexure test.

Sample	*δ* (mm) (±6%)	*P* (N) (±5%)	*σ_f_* (kPa)	*ε_f_* (%)
SAC	9.37	11.72	210.6	18
FSC-20MG	5.52	9.34	167.4	9
FSC-80MG	4.78	10.48	189.0	8
CAC	10.30	9.91	178.2	17
HSC	9.14	22.36	401.4	34

**Table 5:** Geometric parameters of fabricated aerogel composites.

Sample *	Planar Dimension (mm^2^)	Thickness (mm)	Weight (g)	Density (kg/m^3^)
SAC	101.7 × 101.6	9.8	10.63	104.97
FSC-20MG	101.8 × 101.9	9.9	12.16	97.99
FSC-80MG	101.8 × 101.9	9.9	10.99	107.01
CAC	102.4 × 102.5	9.8	10.6	103.05

* Note: SAC = Silica Aerogel Composites; FSC =Fumed Silica Composites; CAC = Carbon nanotube Aerogel Composites.
